# Role of Technology in Detection of COVID-19

**DOI:** 10.7759/cureus.29138

**Published:** 2022-09-13

**Authors:** Drishti V Lohiya, Swanand S Pathak

**Affiliations:** 1 Medicine, Jawaharlal Nehru Medical College, Datta Meghe Institute of Medical Sciences, Wardha, IND; 2 Pharmacology, Jawaharlal Nehru Medical College, Datta Meghe Institute of Medical Sciences, Wardha, IND

**Keywords:** cas system, thermometer thermal scanners, artificial intelligence, elisa, serological test, molecular investigations, ct-scan, rt-pcr, covid, sars-cov-2

## Abstract

The severe acute respiratory syndrome coronavirus 2 (SARS-CoV-2) virus caused coronavirus infection termed as COVID-19, an illness that has spread devastation all over the world. It was developed first in China and had swiftly spread throughout the world. COVID has created imposed burden on health in the lives of all individuals around the globe. This article provides a number of unprecedented detection technologies used in the detection of infection. COVID has created a large number of symptoms in the young, adolescent as well as elderly population. Old age people are susceptible to fatal serious symptoms because of low immunity. With these goals in mind, this article includes substantial condemning descriptions of the majority of initiatives in order to create diagnostic tools for easy diagnosis. It also provides the reader with a multidisciplinary viewpoint on how traditional approaches such as serology and reverse transcriptase polymerase chain reaction (RT-PCR) along with the frontline techniques such as clustered regularly interspaced short palindromic repeats (CRISPR)/Cas and artificial intelligence/machine learning have been utilized to gather information. The story will inspire creative new ways for successful detection therapy and to prevent this pandemic among a wide audience of operating and aspiring biomedical scientists and engineers.

## Introduction and background

COVID-19 is a fatal illness caused by the virus, severe acute respiratory syndrome coronavirus 2 (SARS-CoV-2), which produces a large number of symptoms. The pandemic originated in China in December 2019 and had completely disseminated worldwide. In spite of several efforts to eradicate the disease, it continued to exist in a large number of nations with large levels of clinical manifestations [1,2]. A multimodal approach including correct diagnosing and monitoring for prevention was essential to control this pandemic. On the other hand, timely diagnosis using advanced technologies was critical. With the rise in the number of COVID cases estimated, SARS-CoV-2 identification was crucial for successfully preventing and treating COVID-19 cases as well as detecting virus spread. The best standard for identification is the reverse transcriptase method [3].

SARS-CoV-2 leads to a number of stumbling blocks to healthcare facilities and medical infrastructure as a result of its unprecedented global spread. Early diagnostic devices, effective medical care processes and utmost important disease inoculations are all instantly required according to the international research community. A vital need of the hour was to bring together experts from various fields to develop innovative solutions. New principles for quick advanced diagnosis, detection and treatment have all emerged as a result of several cutting-edge technologies [4].

## Review

Digital health technologies can help in finding easy strategies and responses to the pandemic as compared to manual methods that are strenuous to implement. As an example, South Korea has combined online technology with government-aided containment and alleviation measures such as monitoring, testing, contact tracing and rigorous isolation which can be linked to their previous flat incidence curves. Despite having just 0%-5% of COVID mortality per 100,000 individuals, the United States had 10 times the number of deaths per capita, has three times the number of censorious care beds per 100,000 people and has been in the rank of number one in epidemic preparations earlier to the COVID-19 pandemic [5].

COVID can be detected by reverse transcriptase polymerase chain reaction (RT-PCR) in India very easily and precisely. In uninfected patients, different vaccines are utilized to prevent the disease. Molecular investigations, serology testing and CT scans are among all other investigations used to get a complete picture of the healing process. Prior screening and detection of coronavirus infection often involve the use of nasopharyngeal and/or oropharyngeal swabs, bronchoalveolar lavage fluid, sputum, bronchial aspirate or blood [6]. SARS-CoV and the Middle East respiratory syndrome-related coronavirus (MERS-CoV) are two viruses that cause upper and lower respiratory tract infections and have similar prior and basic signs and symptoms, making coronavirus infections difficult to differentiate from many other bronchial illnesses. As a result, laboratory testing is particularly significant in addition to clinical and epidemiological investigation [3].

Real-time reverse transcriptase polymerase chain reaction (rRT-PCR)

PCR is a highly sensitized laboratory technology that in the past time is known to work in various biological aspects, capitulating both qualitative and quantitative findings. Real-time reverse transcriptase polymerase chain reaction (rRT-PCR) is a diagnostic qualification PCR used to detect targeted RNAs in clinical samples for infection detection in molecular diagnostics laboratories. The SARS-CoV-2 genome was detected in biological materials using rRT-PCR during the COVID-19 epidemic. Despite its moderate sensitivity and high specificity, the Centers for Disease Control and Prevention (CDC) and World Health Organization (WHO) consider it to be the best-standardized test for coronavirus confirmation [7].

To identify the presence of coronavirus disease (CoV), the RT-PCR test technique was recognized as a highly specified messenger RNA detecting approach. Researchers can identify the suspects using the RT-PCR assay which employs fluorescent dyes. The primer that selects out the viral nucleocapsid genes (N1 and N2) was created by the center in the United States, and the WHO's test selects out the viral RNA-dependent RNA polymerase gene and E gene [1,8]. But RT-PCR slows down the illness detection process since it needs many reagents, experienced labor and specialized equipment [9]. Chest CT scans have lower diagnostic sensitivity than rRT-PCR; thus, everyone exhibiting suspected symptoms must have rRT-PCR. As the sensitivity and specificity is not 100% accurate, it is necessary to perform the test three times in order to completely rule out COVID-19 because the initial negative rRT-PCR result is not always able to do so [10].

Computed tomography (CT) scan

**Figure 1:**
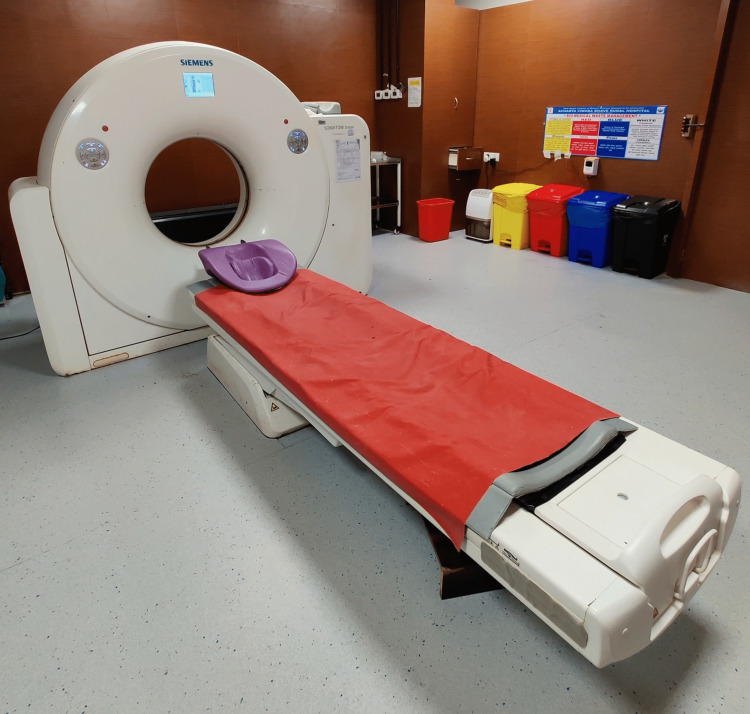
Computed Tomography (CT) Scan Image credit: Author Drishti Lohiya.

Although RT-PCR is still advised for diagnosing COVID-19, the idea of a quicker and more precise diagnostic technique is worth exploring [11]. In the initial examination of the COVID patients' population, a non-contrast chest CT proves to be beneficial. The major professional scientific societies like American Medical Association (AMA) and British Medical Association (BMA) are still not convinced by the significance; the Fleischner Society outlined three instances in which imaging could be utilized as a major diagnostic tool in a recent statement. Patients with regular COVID-19-like respiratory symptoms but no risk pertaining to illness progression, patients with medium to acute COVID symptoms irrespective of RT-PCR test reports and those diseased with medium-to-high symptoms in prone red zone areas were all investigated [12]. Figure 1 shows the currently used CT machine in various health sectors.

The SARS-CoV virus is a Coronaviridae class of organism that can cause systemic and/or respiratory infections and also a multiorgan failure in rare cases. The angiotensin-converting enzyme (ACE) is in charge of the virus cellular entrance. Ground-glass opacity (GGO) is the most commonly seen anomaly in people with COVID-19-related pneumonia, unifocal at first but is frequently multifocal, bilateral and peripheral with a posterior predominance, especially in the inferior lobes of the lungs. Arterial hypertrophy and traction are two common GGO effects that can be studied under CT. COVID-19 bronchiectasis a condition in which the lung airways are damaged which cause difficulty in the clearing of mucus can also be easily seen via CT scan [6].

The CT scan can show the required result up to four days after the first symptom appears (0-4 days). The CT results alter over time in a predictable pattern. The person infected with the disease shows changes for five to eight days. Inside the GGO, thicker interlobular and intralobular lines emerge seldom and the afflicted areas typically expand. "Crazy paving" is the term for this design. Because it is not found in other viral pneumonia, it can help limit the list of options [13].

Clustered regularly interspaced short palindromic repeats (CRISPR)/Cas system

New detection tools worked on the clustered regularly interspaced short palindromic repeats/Cas (CRISPR-Cas) system, which with its better detection accurate measures, has come up for better diagnosis [1,14]. Genomic research, genome editing, gene therapy and genome mapping have all been transformed by the RNA-guided endonuclease (RGEN)-based CRISPR/Cas system [15]. Adaptive immunity systems opposed to bacteria and archaea are the CRISPR and Cas proteins. The CRISPR loci are made up of a CRISPR array and a special spacer sequence (between 30 and 40 bases) [16]. Numerous Cas genes that encode the CRISPR system's effector enzymes were found near the CRISPR array. CRISPR systems can be used efficiently as molecular diagnostics for the detection of nucleic acid and show significant detection efficiency within 30-60 minutes. Three stages make up CRISPR-mediated immunity: adaptation, maturation of the CRISPR RNA (crRNA) and interference [9].

The approach called specific high-sensitivity enzymatic reporter unlocking (SHERLOCK) was first introduced in 2017 and has since made minor advancements. The newest and most advanced version is known as SHERLOCK v2. In addition to Csm6, which shows RNase activity by a trigger of Cas13 nucleases, SHERLOCK also utilizes a variety of other Cas13 enzymes [17]. Accurate multiple sequence detection is made possible by the combination of all these nucleases. Whether the patient's collected sample is positive for viral antigen or not depends on the clinical significance of this technique for detection and diagnosis; identification of the presence of COVID-19, dengue, zika fever or any other viral infection is made possible. For instance, any type of nucleic acid sequence can be found using the SHERLOCK methodology [8].

Pulse oximeter

In order to reduce the risks of severity in affected patients, solution is to have those detection techniques for COVID-19 to educate asymptomatic patients for monitoring their arterial O_2_ saturation by pulse oximetry at home and get themselves diagnosed for care when they show the symptoms of hypoxemia [18]. Throughout the time of this outbreak, home pulse oximetry proved to be extremely helpful in identifying people who need oxygen and hospitalization. A project led by Cleveland University Hospital is presently on a COVID campaign that promotes the use of disposable wireless sensor worn on the finger that may be used at home to measure peripheral capillary oxygen saturation (SpO_2_). A committed command center receives measurements and notifications from the user's smartphones. Self-regulating cheap internet-purchased small medical-grade pulse oximeters could be a realistic solution. Pulse oximetry is currently considered routine in preoperative care. It is also frequent in emergency departments and intensive care units. In today's society, no one can deny evidence-based medicine's utility or clinical benefit [19].

Digital thermometer and thermal scanners

**Figure 2:**
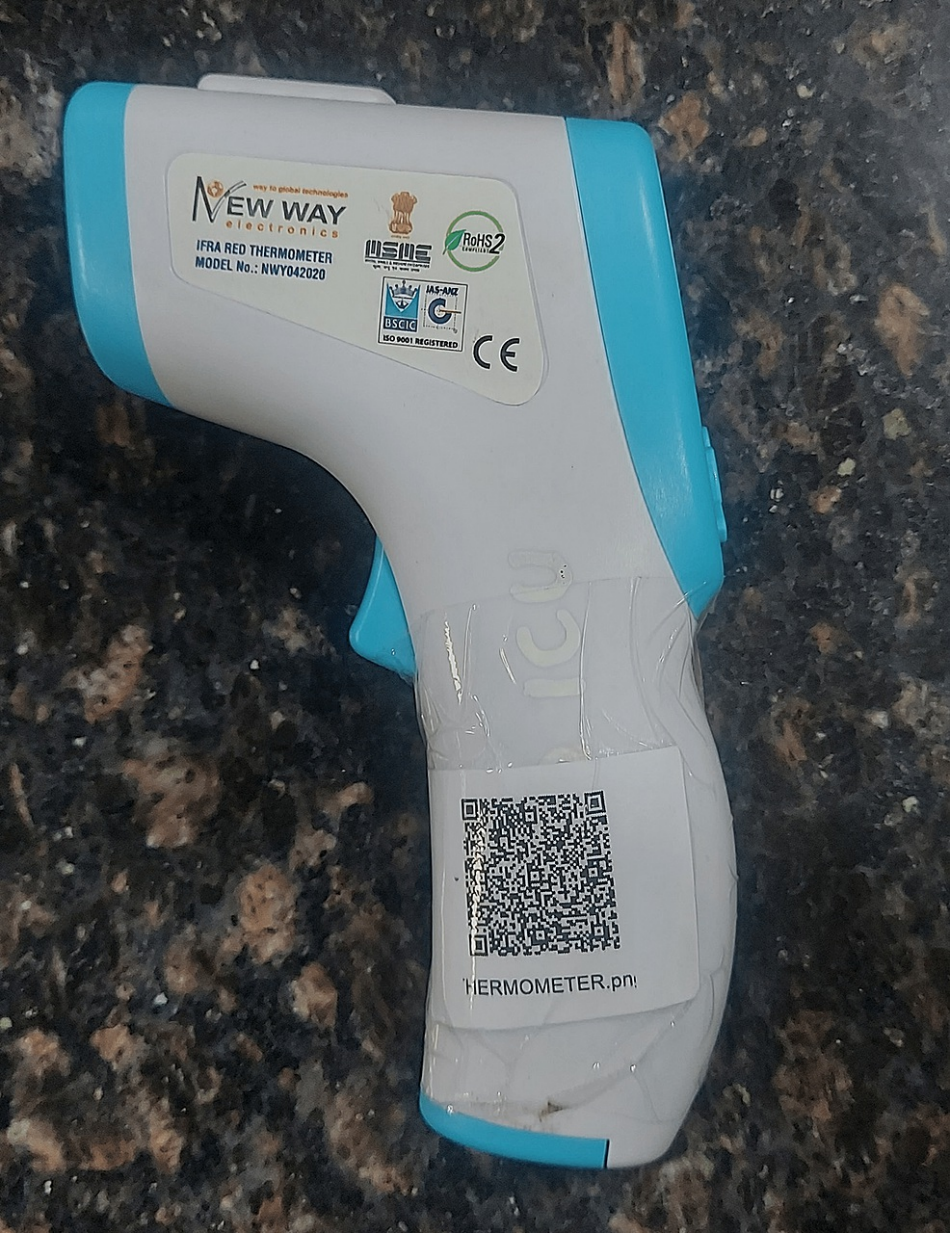
Thermal Scanner Image credit: Author Drishti Lohiya.

Several countries adopted various control measures in response to the SARS virus's appearance in 2003. The safe and very precise method to measure body temperature in any malady disease eruption like SARS, H1N12 and the current COVID-19 is thermal screening which uses small, easy-to-handle non-contact infrared thermometers (NCITs) and thermal scanners (infrared thermal imaging equipment). It operates on the principle of the fact that the organisms release infrared rays like other electromagnetic rays, which can be directed toward a detector that can transform heat signals into electrical signals and display the temperature of the surrounding like a graphic profile (thermal scanners) or a numerical reading (NCITs) [20]. Following COVID-19, thermal screening is mostly used in every place in the world. Entry and/or departure screening is present in the places like airways, defense placements, workplaces, vegetable vendors, shopping complexes and restrains [21]. Figure 2 depicts a thermal scanner for temperature detection used on large scale during COVID times.

The success of thermal screening in the course of prevalence would be based on several variables which include the precision of the instruments for detecting fever and the pertinent of fever among those who have been exposed to the disease. This is set out to perform a thorough analysis and meta-analysis to determine the diagnostic efficacy of NCITs and thermal scanners for the diagnosis of fever [21].

Serological tests

Serological tests are used to identify anti-SARS-CoV-2 antibodies as they are opposed to molecular diagnostic testing, which looks for SARS-CoV-2 RNA [22]. Lateral flow immunoassays (LFIAs), enzyme-linked immunosorbent assays (ELISAs) and chemiluminescence immunoassays (CLIAs) are the three serological tests that are most frequently utilized; they help in finding IgM, IgA, IgG or total antibodies depending on the assay [23]. The specific antibodies that differentiate different assays can identify assays that are directed as opposed to the nucleocapsid (N) protein, spike (S) protein or nucleocapsid and spike (NS) proteins [24].

Along with RT-PCR and another nucleic acid tests in the detection of COVID, serological assays have drawn a lot of interest since some may be of less cost and simple to use. These assays have a significant profit over RT-PCR, these can identify people who suffered SARS-CoV-2 infection in the past even if they have never undergone any testing when they were acutely unwell [1]. In a short span of time, numerous serological assays for COVID-19 have been accessible including some that are advertised for use as quick point-of-care diagnostics. However, development has outpaced the rigorous evaluation pace leaving significant test accuracy uncertain [25].

Enzyme-linked immunosorbent assay (ELISA)

Multiple ELISA-based techniques with large duplicability and long-term reactivity exist, making the test a useful tool for diagnosing a wide range of infectious diseases. It takes nearly five hours to perform a qualitative or quantitative test. COVID-19 antigens and human antibodies are opposed to COVID antigens, which are identified using sandwich and indirect ELISA methods. Epitope Diagnostics Incorporation recently developed an ELISA kit based on IgG and IgM to diagnose SARS-CoV-2 (EDITM Novel Coronavirus COVID-19 ELISA Kit). The National Institute of Virology in Pune, India, initiated the establishment of an indigenous IgG-based ELISA for COVID-19 antibody diagnosis in conjunction with Zydus Diagnostics (COVID KAVACH ELISA). The COVID-19 (COVID KAVACH ELISA) antibody detection has been tentatively validated [26]. The COVID KAVACH ELISA exhibited high reactivity and particularity for the diagnosis of SARS-CoV-2 infection during preliminary validation. Other ELISA kits for detecting coronavirus infection by evaluating immunoglobulin A and immunoglobulin G antibodies are available in addition to the ones described above [3].

Lateral flow immunoassay (LFIA)

Since virus enables quick discernment of molecular ligands in substrates, lateral flow immunoassay (LFIA)-based technologies referred to as immunochromatographic tests have been successfully used in some parts of the world for the past 60 years in the diagnosis of numerous diseases and conditions. These diagnostic platforms are becoming more and more common in hospitals especially those with limited funds and staffing as well as in homes for personal health monitoring. These inexpensive tools have advantages over contemporary lab-based analyzers due to their accessibility, capacity for quick detection and simplicity of use. These portable diagnostic equipment have high analytical responsiveness and accuracy along with the straightforward visual readout of data that are principally responsible for its allure [27].

The relevance of particular viral antigens such as the spike (S) protein or the nucleocapsid (N) protein using a technique called lateral flow immunoassay (LFIA). These antigen tests are designed to carry out population-wide screening for epidemiological uses which helps to check for the scope of the virus's distribution in a neighborhood as well as to swiftly authenticate the existence of SARS-CoV-2 in nasopharyngeal swabs from individuals exhibiting recognizable symptoms. When someone is tested in the period of the high viral prone area and when exposed to a COVID-19 carrier, it is checked that the best findings from LFIAs are frequently achieved [28].

Chemiluminescence assays (CLIAs)

As of May 31, 2021, the SARS-CoV-2-induced 2019 coronaviruses illness (COVID-19) has led to a majority of 170 million long-standing cases and the highest of 3.53 million fatalities. SARS-CoV-2 encodes nonstructural and auxiliary proteins with the four main structural proteins, spike (S), envelope (E), membrane (M) and nucleocapsid (N). Virus adherence and host cell invasion are mediated by the spike protein (S protein) on the surface of the virus cover [1]. There are two S1 and S2 subunits that together make up the S protein. The virus enters target cells through the receptor angiotensin-converting enzyme 2 (ACE2) through its receptor-binding domain (RBD). S1 binds to ACE2 on the host cell changing its conformation so that S2 can fuse with the membrane of the host cell [29].

Chemiluminescence assays (CLIAs) which combine immunochemical reactions and chemiluminescence technology can be used to measure the levels of IgG and IgM antibodies. Using chemiluminescence technology, various proteins and immunoglobulins were created. These techniques were tested on 86 SARS-CoV-2 vaccine contributors sera and 119 COVID-19 convalescent patients sera. By comparing the four approaches to the neutralization assay, the four detection methods were assessed to check the ability of neutralizing antibodies in the serum of COVID-19 diseased and immunized beneficiaries [30].

Artificial intelligence (AI)

The practice of using currently available medications to treat new and difficult-to-treat disorders such as COVID-19 is known as drug repurposing or repositioning. Due to the potential for less development time and lower cost management, drug repurposing has evolved as a successful strategy. Artificial intelligence (AI) and network medicine provide frontline informatic science applicable to define disease, medicine, therapies and discover targets with very few mistakes in the age of big data. AI models can hasten COVID-19 drug repurposing. Artificial intelligence and network medicine technologies that are powerful, innovative and quickly developing can hasten the discovery of new treatments [31]. There are various sectors of the healthcare system where artificial intelligence (AI) is being used which include prognosis, community healthcare, clinical decision-making and treatments. In particular, artificial intelligence algorithms are important for the quick recognition of COVID-19 disease during the ongoing pandemic [32].

Various apps were developed in various countries to detect carriers of COVID-19 illness. Aarogya Setu is an official mobile application used by the Government of India for tracing the COVID-19 outbreak and restricting the spread of the disease. Aarogya Setu is a free smartphone application that the Indian Government recently released in 11 Indian languages for the Android and iOS platforms for digital contact tracing of COVID-19 cases. Aarogya means "health" and Setu means "bridge to" in Hindi. This application uses a global positioning system (GPS) and Bluetooth technologies to trace the data. The app uses the Bluetooth technique for communication with all nearby devices. It uses GPS to track the user’s location compared to other smartphone users who have registered as infected and are utilizing a similar application. Such an initiative would have a strong foundation because of the large amount of more than 500 million smartphone users in the nation. The government guarantees privacy to the application users, and no private user data are made available to the general public or third parties. The user's 10-digit Indian contact number and a few personal details are requested when the application is first installed [33]. Along with this, the app also provides additional functions such as self-assessment tests, test reporting, e-permits for traveling, COVID-19-related information, information about precautionary methods and online consultations [34].

In India right now, the "Aarogya Setu" is used by about 100 million people, creating connections between "Aadhaar" the biometric database of 1.22 billion Indian citizens. In the post-COVID-19 era, "Aarogya Setu" has enormous potential for facilitating international travel, social mapping and containing any catastrophic pandemic disasters. However, this potential depends on ensuring public confidence and transparency [33].

Gene sequencing for diagnosis

Viral genome sequencing is not ideal for early detection in large populations due to its high cost, extensive data analysis and limited clinical efficacy when compared to RT-PCR. But utilizing metagenomic RNA sequencing techniques, the first most precise SARS-CoV-2 genome sequence was obtained. The benefit of sequencing-based detection is that viral alterations may be monitored by gathering data on new strains of the virus. Over time, the viral genome can be sequenced to help discover and categorize new coronavirus strains. Based on evidence from closely monitoring the viral evolution, the virus undergoes random mutations in its genome at a rate of about two per month as it multiplies and spreads. Alpha (B.1.1.7), beta (B.1.351), gamma (P.1) and delta (B.1.617.2) are some of the new mutant viruses that have been discovered, which carry the possibility of the virus spreading significantly more quickly. Because of the increasing demand, portable rapid sequencing or high-throughput methods have developed as diagnostic tools for COVID-19 [35].

COVID detection

The knowledge obtained from the detection of other deadly viruses exceptionally SARS-CoV and MERS-CoV was critical in allowing to quickly and precisely diagnose the COVID-19 infection. The SARS-CoV-2 genome sequence was performed in a very short time which is about a month, leading to the increased growth and application of nucleic acid amplification tests (NAATs) as the primary technique of COVID-19 detection which leads to the prior development in the resistance against the virus. Serological tests had also been received a lot of interest since they are more user-friendly and provide more information about all infected people than NAATs. Newer techniques and methodologies that had also come into play for diagnosing COVID-19 infection have been analyzed based on the principles of operation and the value they bring to their viral and throwback detection because diagnostic procedures are a very important aspect of working out the outbreaks; it is critical to address present methods shortcomings, development of well-organized methods and find all infection-spreading people as earlier and properly even if they are asymptomatic COVID-19 carriers. With this goal in mind, lateral flow assays (LFAs), paper-based approaches, microfluidic modules and piezoelectric devices are also some of the useful strategies for prevention, control of the spreading of COVID-19 and any other future pandemic flare-up [1]. To a little extent, these techniques can help in meeting the urgency for point-of-care (POC) and megaplex testing as well as they help to prevent new pandemic outbreaks by making it easier to detect them and faster to receive results from clinics and small healthcare institutions [36].

During the COVID-19 outburst, digital technology has undoubtedly been critical for reducing and minimizing social, physical and psychological risk factors as well as managing the term significance of social isolation and lockdown loneliness. However, because the reverberation of social separation and lockdown isolation take time to appear, most people who were affected by the pandemic may not feel separated straight. As a result, digital technologies must not only contribute to social connectivity and minimal lockdown separation but also should provide a person at the peril of loneliness to prevent communal isolation in the course of and after the COVID-19 outbreak. Cost and competence of experts is one of the most critical issues to be addressed. For overcoming separation, eager collaborators who use digitized technology necessarily would come a lot into play [37,38].

Many potential medical treatments, vaccinations and technological advancements are being studied by researchers worldwide to combat COVID-19. However, there is still a lack of evidence that vaccines can keep people completely safe, so in addition to vaccine production, therapeutic medicine research deserves special focus. Blocking the stages of the viral lifecycle can provide a potential cure for infection [39].

## Conclusions

To conclude the given review, technology is a very necessary factor that plays a very important role in the process of disease detection, control and management. Without the proper technologies for detection of COVID-19, treatment would not have been possible. Various detection tools like rRT-PCR, CT-SCAN, CRISPR and ELISA are very important in the diagnosis of disease through various process of detection techniques not only in COVID-19 pandemic but also in many other disease detection. Without all these detection techniques, COVID treatment would have been impossible. All these detection tools are very important to save lives not only from COVID infection but also from various other infectious diseases. The inventions of all these techniques had helped a lot in building science infrastructure. Not only the detection of COVID-19 infection but also technology has a lot more to do with the vaccines and drug development for the prevention and treatment of the illness. Technology had played a very crucial role in the enhancement of vaccine preparation process at a huge level. Various types of vaccines are prepared against COVID-19 as live-attenuated coronavirus vaccine, recombinant COVID-19 vaccines like nucleic acid-based coronavirus vaccine, protein-based coronavirus vaccine, vectored vaccines against coronavirus, etc. Along with all these types, mRNA vaccine against COVID-19 is also developed with the use of technology which is considered a very effective vaccine against the infection. Drug development against COVID-19 also could not be possible without the use of technology.
